# Polyethylene terephthalate clamps: Optimization in endodontic and restorative practices

**DOI:** 10.4317/jced.59603

**Published:** 2022-08-01

**Authors:** Anna-Flávia Ferreira e Cunha, Isabela-Ribeiro Madalena, Erika-Calvano Küchler, Tiago-Lima Pereira, Richard Honorato, Fernando-Carlos-Hueb de Menezes, César-Penazzo Lepri, Maria-Angélica-Hueb-de Menezes Oliveira

**Affiliations:** 1 Department of Biomaterials, University of Uberaba - UNIUBE, Uberaba, Minas Gerais, Brazil; 2 Department of Dentistry, University of Joinville Region, Joinville, Santa Catarina, Brazil; 3 School of Dentistry, Presidente Tancredo de Almeida Neves University Center, São João del Rei, Minas Gerais, Brazil; 4 Department of Restorative Dentistry, School of Dentistry, Federal University of Juiz de Fora, Juiz de Fora, Minas Gerais, Brazil; 5 Department of Orthodontics, University of Regensburg, Regensburg, Germany

## Abstract

**Background:**

There is a growing search for innovations in dental materials and instruments and, therefore, an increase need to optimize the instruments used in the absolute isolation. The gold standard procedure contributes significantly to the quality of restorative and endodontic procedures. Thus, the aim of the present study was to evaluate the radiopacity of polyethylene terephthalate polymer clamps and compare them to conventional metal clamps.

**Material and Methods:**

The polyethylene terephthalate clamp was developed at the University of Uberaba (Patent application #PI0901719-4, Uberaba, MG, Brazil). Five polyethylene terephthalate clamps and five conventional metal clamps were used. The clamps were positioned, next to an aluminum scale, under the same phosphor plate to perform 3 radiographs. The locator cylinder was set perpendicular to the radiographic films at a focal length of 20 cm and set to 60 kVp and 0.06 seconds. After image processing, optical density values were read using DBWin 5.0.4 software. The mean of the 3 readings taken on each clamp was adopted as the radiodensity of the specimen. The differences between the groups were compared using Student’s t-test (*p*<0.05).

**Results:**

Polyethylene terephthalate clamps demonstrated significantly lower radiopacity than conventional metal clamps (*p*<0.05).

**Conclusions:**

Polyethylene terephthalate clamps have lower radiopacity when compared to conventional metal clamps.

** Key words:**Rubber Dams, Dentistry, Operative, Endodontics.

## Introduction

Absolute isolation is essential for the quality and optimization of dental restorative (1,2) and endodontic procedures (3,4). In addition to reducing contamination by microorganisms, oral fluids and cross-infection, they allow for better visibility and safety in the handling of some materials and instruments. Furthermore, in pediatric dentistry practice, we can still point out that absolute isolation contributes to the management of child behavior (5,6). The technique eliminates the discomfort of cotton rolls in addition to minimizing the sensation of invasion felt by the child in some procedures.

Several techniques for absolute isolation have been proposed (7-9). Absolute rubber dam isolation is still the gold standard technique for all types of procedures (7,10). It can be said that the choice of the ideal clamp is directly proportional to the optimization of the technique (7). Thus, the performance of metal clamps widely used (7,10). The metal clamps allow dam stability and even retraction of the gingival tissue (11). However, disadvantages such as injuries to dental and gingival tissues, intraoperative sensitivity, difficulty in handling, cost and influence by overlapping images have also been mentioned (9,11-13).

 Scientific evidence has proposed clamps and adaptations in order to reduce injuries to dental and gingival tissues as well as intraoperative sensitivity (7,11,14). Polymer clamps were proposed because they are more malleable and facilitate adaptation (8,15,16). It is worth noting that radiopacity and cost still prevail in these clamps types. Given the above, it is assumed that polymer clamps developed from polyethylene terephthalate may be a viable option. In addition to radiolucency, polyethylene terephthalate is inexpensive and readily available (17,18). Thus, the aim of the present study was to evaluate the radiopacity of polyethylene terephthalate polymer clamps and compare them to conventional metal clamps.

## Material and Methods

-Experimental design

Polymer clamps developed at the University of Uberaba (patent application #PI0901719-4) and conventional metallic camps commercially available and commonly used in clinical practice were evaluated in this study, as listed in Table 1. Five clamps of each material were used for radiopacity analysis.

**Table 1:**
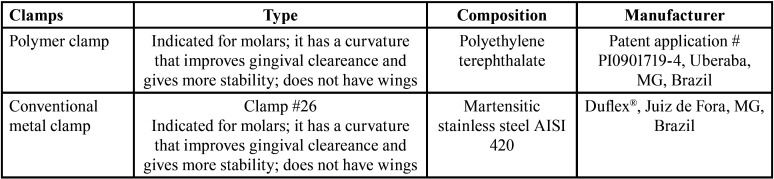
Clamps characteristics.

-Analysis of Radiopacity 

The Siemens Heliodent 60B X-ray machine (São Paulo, SP, Brazil) and the VistaScan Mini Easy system (Dürr Dental, Bietigheim-Bissingen, Germany) were used to obtain the radiographic images in this study. The clamps of both compositions were positioned on a phosphor plate next to a 12-level aluminum scale. The aluminum scale ranges from 1 to 12 mm and considers the zero level to be a direct exposure on the phosphor sensor (100% of the emitted radiation). The locator cylinder was set perpendicular to the radiographic films at a focal length of 20 cm and set to 60 kVp and 0.06 seconds . Each specimen was exposed to ionizing radiation simultaneously for 3 times (19).

Optical density values under the images were read using DBSWin Imaging Software 5.0.4 (Air Techniques Inc. Melville, NY, USA). DBSWin Imaging is a software based in Windows. It is capable of measuring density curves of digital radiographs obtained by digital X-rays impregnating on the VistaScan Mini Easy sensor. The density measurement tool automatically measures gray scale values in the image. Only regions that were free of air voids, gaps, cracks or other similar defects were evaluated. A mean of 3 consecutive readings was obtained to evaluate the clamps of each group.

-Statistical analysis

The differences between the groups were compared using Student’s t-test. The significance level was set at 5%.

## Results

Table 2 shows the mean and standard deviation (SD) in pixels of the radiodensity of constituent parts of the polymer clamp and the conventional metal clamp. The percentage of radiation that passed the clamps and reached the phosphor plate is also presented. The polymer clamp is less radiopaque when compared to the conventional staple (*p*<0.05).

**Table 2:**
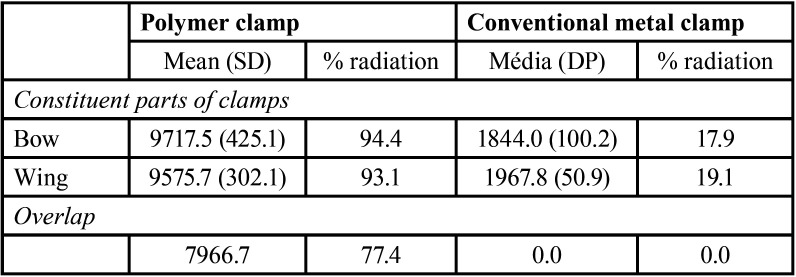
Mean and standard deviation of radiodensity of constituent parts of the polymer clamp and the conventional metal clamp. The percentage of radiation that passed the clamps and reached the phosphor plate is also presented.

**Figure 1:**
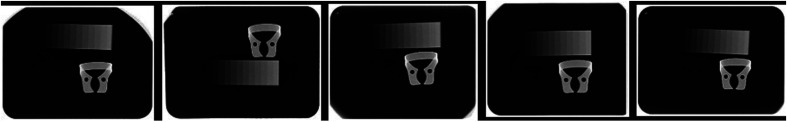
Illustration of the radiographs of the polymer and conventional metal clamps together with the aluminum scale.

Figure 1 illustrates the radiograph of the polymer and conventional metal clamps together with the aluminum scale.

## Discussion

Absolute isolation using a rubber dam and clamps remains the gold standard for performing procedures in dental practice (7,10). However, several disadvantages regarding the difficulty of the technique with conventional metal clamps, patient sensitivity, cost, and overlapping of images, when radiographic complementation is needed, are often highlighted (9,11-13). Some adaptations to conventional metal clamps have already been suggested (8,15,16). In view of the above, the aim of the present study was to evaluate the radiopacity of polyethylene terephthalate polymer clamps and compare them to conventional metal clamps. Our results demonstrate that the polyethylene terephthalate polymer clamp is significantly less radiopaque than conventional metal clamps. This result mainly highlights the ease of obtaining radiographic images.

In order to contribute to innovations in the dental market and provide professionals and patients with comfort and safety during dental practice, the University of Uberaba (UNIUBE) developed a “Device for Absolute Isolation in Invasive Dental Procedures” (patent application #PI0901719-4) which can be easily described by a polyethylene terephthalate polymer clamp. The clamp is placed in the oral environment with the aid of forceps (clamp holder), after placing the rubber dam. The seating tabs adhere completely to the dental element, enveloping it by means of the tabs and/or the auxiliary circular rubber; procedures similar to conventional clamps. (7,10) However, the polyethylene terephthalate polymer clamp developed by UNIUBE stands out from the other clamps available on the market, since it has radiolucency and does not cause overlapping images on the dental tissues.

Radiolucency/radiopacity is an important property of dental materials and instruments. Such characteristics are measured by optical density. The greater the degree of darkening, the greater the density of the material/instrumental and the smaller the amount of light that will pass through the radiograph when placed in front of a negatoscope or light focus (20). By reading the optical density values of the clamps tested in this study, we obtained as a result that conventional metal clamps, due to their high molecular density, absorb all photons, not letting radiation reach the phosphor plate. Such an assertion implies 0.0% of radiation exceeded and the absence of superposition of the image of the metallic clamp, forming a radiopaque image. The polyethylene terephthalate polymer clamp, on the other hand, has a low molecular density, allowing the photons to pass through the material and reach the phosphor plate. 77.4% of the radiation passes through the material, causing an overlap and forming a radiolucent image.

Polyethylene terephthalate is widely produced in textile, packaging, construction, automotive and biomedical industries (17,18,21). Among its main advantages over other polymers, one can cite biocompatibility, excellent thermal and chemical stability, high hydrolytic stability, ease, low processing cost, considerable lightness, and aesthetics (17,18). It can be noted that in terms of thermal stability, polyethylene terephthalate is sTable when heated at temperatures below 230 °C for 50 minutes, which suggests the possibility of sterilization as well as conventional metal clamps. On the other hand, the ease and low cost of processing also imply the hypothesis of disposable use.

It is worth note that another type of polymer clamp is described in the scientific literature (8,15,16). This does not show radiolucency; such a polymer clamp was specially developed to reduce damage to dental and gingival tissues. Because polymers are more flexible, they result in less pressure on the tooth surface and become more comfortable for the patient (10). Even without testing the flexibility of the polyethylene terephthalate clamp and patient comfort, it is suggested that due to the aforementioned properties of the material, characteristics such as these can be achieved.

Finally, the main advantage of the polyethylene terephthalate polymer clamp is highlighted, as it does not prevent the visualization of dental structures and materials arranged under dental tissues; other studies are needed to complement evidence on its adaptation and durability for the optimization of dental practices. 

## Conclusions

Polyethylene terephthalate polymeric clamps show less radiopacity when compared to conventional metallic clamps.
